# Supplementary material for: Causal Relationship between Aging and Anorexia Nervosa: A White-Matter-Microstructure-Mediated Mendelian Randomization Analysis
Source: Biomedicines. 2024 Aug 16;12(8):1874. doi: 10.3390/biomedicines12081874 (PMC11351342; doi:10.3390/biomedicines12081874)
Supplement: Supplementary file 1 [file biomedicines-12-01874-s001.zip › Supplementary Figure/Figure S8/Leaveoneout plot of MR analysis for TL to ubm-b-2118.pdf]

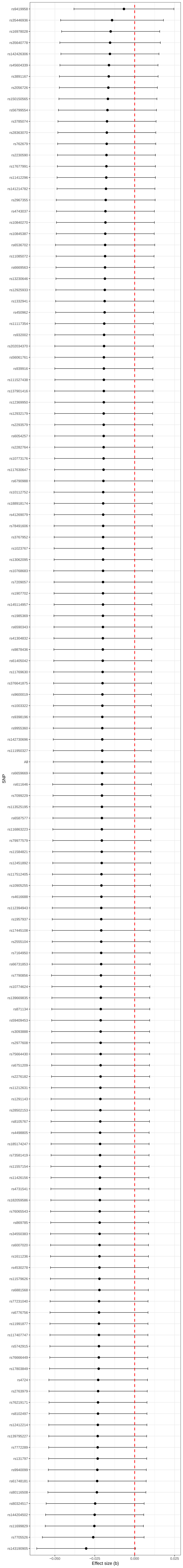
rs9419958
rs35446936
rs16978028
rs35640778
rs142426306
rs45604339
rs3891167
rs2056726
rs150150565
rs56799554
rs3785074
rs28363070
rs762679
rs2230590
rs17677991
rs11412296
rs141214782
rs2967355
rs4743037
rs10840270
rs10845387
rs6536702
rs11085072
rs6669563
rs13230646
rs12925933
rs1332941
rs450962
rs11117354
rs932002
rs202034370
rs56061761
rs939916
rs111527438
rs137901416
rs12369950
rs12932179
rs2293579
rs6054257
rs2282764
rs10773176
rs117630647
rs6790988
rs10112752
rs188918174
rs41269079
rs78491606
rs3767952
rs1023767
rs13062095
rs10768683
rs7209057
rs1907702
rs145114957
rs1985369
rs6590343
rs41304832
rs9878436
rs61405042
rs11769630
rs376641875
rs9600019
rs1003322
rs9398196
rs9955360
rs142730696
rs111950327
A8
rs6659669
rs611646
rs7099229
rs113525195
rs6587577
rs116863223
rs79977579
rs11584821
rs12451892
rs117512405
rs10905255
rs4616688
rs112394943
rs1957937
rs17445108
rs2555104
rs7164950
rs66731853
rs7790856
rs10774624
rs139669835
rs871134
rs59409453
rs3093868
rs2977608
rs75664430
rs6751209
rs2276182
rs11212631
rs1291143
rs28502153
rs8105767
rs4498805
rs185174247
rs73581419
rs11557154
rs11426156
rs4731541
rs182059566
rs76065543
rs869785
rs34550383
rs6007020
rs1611236
rs4530278
rs11579626
rs6881568
rs77231040
rs6776756
rs11991877
rs117407747
rs5742915
rs76666449
rs17803849
rs4724
rs2763979
rs76219171
rs8102497
rs12412214
rs139795227
rs7772289
rs131797
rs9940099
rs61748181
rs80116508
rs80324517
rs144204502
rs11699829
rs7705526
rs143190905
SNP
-0.050
-0.025
0.000
0.025
Effect size (b)
